# Postoperative muscle loss, protein intake, physical activity and outcome associations

**DOI:** 10.1093/bjs/znac384

**Published:** 2022-11-17

**Authors:** Rianne N M Hogenbirk, Willemijn Y van der Plas, Judith E K R Hentzen, Laura van Wijk, Allard G Wijma, Carlijn I Buis, Alain R Viddeleer, Geertruida H de Bock, Cees P van der Schans, Gooitzen M van Dam, Schelto Kruijff, Joost M Klaase

**Affiliations:** Department of Surgery, University Medical Center Groningen, University of Groningen, Groningen, The Netherlands; Department of Surgery, University Medical Center Groningen, University of Groningen, Groningen, The Netherlands; Department of Surgery, University Medical Center Groningen, University of Groningen, Groningen, The Netherlands; Department of Surgery, University Medical Center Groningen, University of Groningen, Groningen, The Netherlands; Department of Surgery, University Medical Center Groningen, University of Groningen, Groningen, The Netherlands; Department of Surgery, University Medical Center Groningen, University of Groningen, Groningen, The Netherlands; Department of Radiology, University Medical Center Groningen, University of Groningen, Groningen, The Netherlands; Department of Epidemiology, University Medical Center Groningen, University of Groningen, Groningen, The Netherlands; Department of Rehabilitation and Health Psychology, University Medical Center Groningen, University of Groningen, Groningen, The Netherlands; Research Group Healthy Ageing, Allied Health Care and Nursing, Center of Expertise Healthy Ageing, Hanze University of Applied Sciences, Groningen, The Netherlands; Department of Nuclear Medicine and Molecular Imaging, University of Groningen, Groningen, The Netherlands; TRACER Europe BV, Groningen, The Netherlands; Department of Surgery, University Medical Center Groningen, University of Groningen, Groningen, The Netherlands; Department of Surgery, University Medical Center Groningen, University of Groningen, Groningen, The Netherlands

## Abstract

**Background:**

Skeletal muscle loss is often observed in intensive care patients. However, little is known about postoperative muscle loss, its associated risk factors, and its long-term consequences. The aim of this prospective observational study is to identify the incidence of and risk factors for surgery-related muscle loss (SRML) after major abdominal surgery, and to study the impact of SRML on fatigue and survival.

**Methods:**

Patients undergoing major abdominal cancer surgery were included in the MUSCLE POWER STUDY. Muscle thickness was measured by ultrasound in three muscles bilaterally (biceps brachii, rectus femoris, and vastus intermedius). SRML was defined as a decline of 10 per cent or more in diameter in at least one arm and leg muscle within 1 week postoperatively. Postoperative physical activity and nutritional intake were assessed using motility devices and nutritional diaries. Fatigue was measured with questionnaires and 1-year survival was assessed with Cox regression analysis.

**Results:**

A total of 173 patients (55 per cent male; mean (s.d.) age 64.3 (11.9) years) were included, 68 of whom patients (39 per cent) showed SRML. Preoperative weight loss and postoperative nutritional intake were statistically significantly associated with SRML in multivariable logistic regression analysis (*P* < 0.050). The combination of insufficient postoperative physical activity and nutritional intake had an odds ratio of 4.00 (95 per cent c.i. 1.03 to 15.47) of developing SRML (*P* = 0.045). No association with fatigue was observed. SRML was associated with decreased 1-year survival (hazard ratio 4.54, 95 per cent c.i. 1.42 to 14.58; *P* = 0.011).

**Conclusion:**

SRML occurred in 39 per cent of patients after major abdominal cancer surgery, and was associated with a decreased 1-year survival.

## Teaser

This prospective observational cohort study aimed to identify the occurrence of and risk factors for surgery related muscle loss (SRML). SRML occurred in 39 per cent of patients within 1 week of major abdominal surgery and was associated with preoperative weight loss, and insufficient postoperative nutritional intake and physical activity. Reduced 1-year survival was seen in patients with SRML within 1 week of surgery.

## Introduction

Clinically relevant surgery-related muscle loss (SRML) refers to the postoperative loss of muscle mass resulting from the stress response induced by major surgical trauma^1,2^. In critically ill patients in the ICU, knowledge of muscle loss, ‘ICU-acquired weakness’, and their long-term consequences is growing^3,4^. However, for patients undergoing major surgery, a few studies have focused on the presence, impact, and possible predictors for clinically relevant SRML^5–8^.

Since abdominal surgery forms the cornerstone of treatment for oncological hepatic, pancreatic, biliary, and colorectal tumours, insight into the physical impact and long-term consequences of major abdominal surgery is essential. Despite advances in surgical techniques and perioperative care, major abdominal surgery places a substantial burden on patients through high morbidity and mortality. However, only a few studies have described an association between surgery-related skeletal muscle loss, postoperative complications, increased duration of hospital stay, decreased quality of life, and poorer long-term survival^5,9–11^.

The primary aim of this study was to establish the incidence of clinically relevant SRML within 1 week of major abdominal surgery and to determine its related risk factors. Furthermore, the long-term consequences of SRML on fatigue and 1-year survival were investigated. With this knowledge, future intervention studies can focus on the prevention of postoperative muscle loss.

## Methods

### Design

This prospective, observational, cohort study was conducted at the University Medical Center Groningen (UMCG) under the name ‘MUSCLE POWER STUDY’^12^. Adult patients with a (suspected underlying) malignant disease who were scheduled for major open abdominal surgery between May 2019 and June 2021 were included. Owing to hospital policy limiting hospital access during periods with a peak incidence of COVID-19, the inclusion of patients was paused between February 2020 and September 2020.

The study protocol was approved by UMCG’s medical ethics committee (METc2018/361, version 3.0, 21 January 2019), registered within the International Clinical Trials Registry Platform (201800445, NL7505, version 1.0, 7 February 2019), and was published previously^12^. The written consent of all participating patients was obtained prior to inclusion. The study was performed in accordance with the ethical standards laid down in the 1964 Declaration of Helsinki and its later amendments.

### Endpoints

The primary endpoint of this study was the number of patients presenting with clinically relevant SRML within 1 week of major abdominal surgery. One secondary endpoint was an association between six possible, predefined predictors and SRML: age 65 years or older; preoperative diabetes mellitus; preoperative sarcopenia; major postoperative complications (Clavien–Dindo grade III or higher); insufficient postoperative physical activity; and insufficient postoperative protein intake. Another secondary endpoint was the long-term consequences of SRML in terms of fatigue and 1-year postoperative survival.

### Data collection

#### Demographics and surgical details

Demographics and surgical details were prospectively collected from the patients’ charts. Preoperative weight loss within the previous 6 months and risk factors for malnutrition were assessed by the Patient-Generated Subjective Global Assessment Short Form (PG-SGA SF)^13^. Surgical parameters included type of operation, duration of operation, estimated blood loss, and pathological resection status (R0 indicates microscopically margin-negative resections; R1 indicates removal of macroscopic disease but with microscopic-positive tumour margins; R2 indicates macroscopic residual tumour that was not resected). Postoperative surgical parameters were duration of hospital stay and complications up to 30 days after discharge clustered according to Clavien–Dindo grades (with major complications defined as grade III or higher) and the Comprehensive Complication Index (CCI)^14,15^.

#### Muscle measurements

Measurements of muscle thickness as a derivate of muscle mass were performed by three researchers (R.N.M.H., W.Y.P., and J.E.K.R.H.) who were trained by a musculoskeletal radiologist (A.R.V.). They used bedside point-of-care ultrasound (POCUS; Philips FUS6882 Lumify L12-4, Koningklijke Philips N.V., the Netherlands) according to a predefined standardized protocol^12^. Patients were positioned lying in supine position on the bed with their arm and leg muscles relaxed. The anteroposterior diameter of the biceps brachii, rectus femoris, and vastus intermedius was measured three times bilaterally at baseline (1 day preoperatively) and 7 days postoperatively. Measurements of the biceps brachii were performed at two-thirds of the length between the acromion and elbow fold, with the elbow extended and the forearm in supine position. Leg muscle measurements were performed halfway between the spina iliaca anterior superior and the proximal border of the patella. Points of measurement were marked with a waterproof marker, to ensure fixed points of measurements. The transducer was placed perpendicular to the long axis of the different muscles (that is, perpendicular to the major axis of the limb). A reduction of 10 per cent or more in muscle thickness in at least one arm muscle and one leg muscle on postoperative day 7 was considered to be clinically relevant SRML.

#### Physical activity and nutritional intake

Physical activity was assessed using the number of steps taken daily, measured by a wearable mobility tracker device on the patients’ ankle for the seven consecutive days postoperatively (wGT3X-BT; Actigraph, Pensacola, Florida, USA). The Actigraph device did not provide feedback to the patient. Step counts were assessed separately for each day and cumulatively according to the total steps taken in the first five postoperative days^16^.

Nutritional intake was assessed as grams of protein consumed per kilogram of body weight per day. All nutritional intake through oral, enteral, and parenteral nutrition was recorded in a nutritional diary for the first seven consecutive postoperative days. Daily protein intake was calculated with a nutrition calculator application (Mijn Eetmeter; Stichting Voedingscentrum Nederland, Rotterdam, the Netherlands). Protein intake was assessed separately for each day and cumulatively according to the total intake in the first five postoperative days.

Insufficient physical activity or nutritional intake was defined as physical activity (steps per day) or nutritional intake (in g/kg body weight) below the lowest tertile for at least 3 days in the first postoperative week.

#### Long-term consequences of surgery-related muscle loss

The 2003 Dutch version of the Multidimensional Fatigue Inventory (MFI) was used to assess fatigue at baseline prior to surgery and 3 and 6 months postoperatively^17^.

Follow-up survival data were collected from the patients’ electronic charts.

### Statistical analysis

SRML was assessed on the seventh postoperative day or, if patients were discharged on the sixth postoperative day, on the day of discharge. When patients were discharged before the sixth postoperative day, they were excluded from analysis. When patients were discharged within seven postoperative days, measurements of nutritional intake and physical activity were assessed up to 1 day prior to discharge.

Demographics and surgical details are presented as mean (s.d.), median (interquartile range (i.q.r.)), or count (%). Independent samples Student’s *t* tests, Mann–Whitney *U* tests, Pearson’s χ^2^ test, and Fisher’s exact test were used to compare patients with and without clinically relevant SRML.

Six possible predefined predictors for clinically relevant SRML (age 65 years or older, diabetes mellitus, preoperative weight loss within 6 months, major complications (Clavien–Dindo grade III or higher), insufficient physical activity, and insufficient protein intake) were assessed by use of univariate and multivariable logistic regression analyses^12^.

The impact of SRML on 1-year survival was investigated using a Cox proportional hazard model adjusted for factors with known associations with postoperative survival (age, type of cancer-specific resection, resection margin status (R0, R1, or R2 resection), and complications in CCI).

## Results

### Demographics

**Fig. 1:**
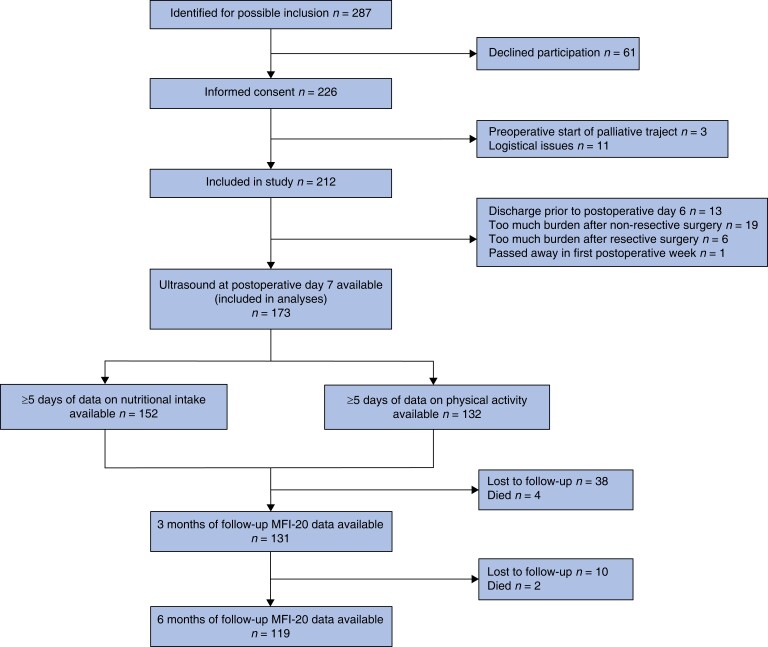
Flowchart of inclusion of the MUSCLE POWER STUDY Overview of assessment of eligibility, inclusion and follow-up of patients in the MUSCLE POWER STUDY. MFI, Multidimensional Fatigue Inventory.

A total of 287 patients were assessed for eligibility, of whom 173 (60.3 per cent) were included in the final analyses. A flow chart of patient inclusion for the MUSCLE POWER STUDY is presented in *Fig. 1*.

A total of 68 patients (39.3 per cent) showed a reduction of 10 per cent or more in diameter of at least one arm muscle and one leg muscle at postoperative day 7 and were, therefore, considered to be patients with clinically relevant SRML. The characteristics and surgical details of patients with and without SRML are presented in *Tables 1 and 2*, respectively.

**Table 1:** Demographics of patients with and without surgery-related muscle loss (SRML)

	Total (*n* = 173)	SRML (*n* = 68)	No SRML (*n* = 105)	*P* value
Age (years)	64.3 (11.9)	65.0 (12.0)	64.0 (11.9)	0.658*
Male sex	95 (54.9)	44 (65)	51 (48.6)	0.037†
Preoperative BMI (kg/m^2^)	26.2 (4.8)	26.0 (4.5)	26.4 (5.0)	0.427*
ASA grade ≥ III	45 (26.0)	20 (29)	25 (23.8)	0.412†
PG-SGA SF ≥ IV	60 (34.7)	25 (38)	35 (33.3)	0.643†
**Preoperative weight loss in 6 months**				0.087†
ȃ<5%	111 (64.2)	39 (57)	72 (68.6)	
ȃ5–10%	28 (16.2)	10 (15)	18 (17.1)	
ȃ>10%	34 (19.7)	19 (28)	15 (14.3)	
**Presence of comorbidities**				
ȃAny comorbidity	107 (61.8)	44 (65)	63 (60.0)	0.534†
ȃCardiac	43 (24.9)	20 (29)	23 (21.9)	0.264†
ȃDiabetes mellitus	21 (12.1)	6 (9)	15 (14.3)	0.283†
ȃHypertension	49 (28.3)	22 (32)	27 (25.7)	0.344†
ȃPulmonary	23 (13.3)	9 (13)	14 (13.3)	0.985†
ȃRenal	6 (3.5)	4 (6)	2 (1.9)	0.163‡
**Prior oncological treatment**				
ȃNeoadjuvant chemotherapy	48 (27.7)	20 (29)	28 (26.7)	0.694†
ȃNeoadjuvant radiotherapy	32 (18.5)	17 (25)	15 (14.3)	0.076†
ȃNone	118 (68.2)	44 (65)	74 (70.5)	0.426†

Data are presented as mean (s.d.) or *n* (%). *Student’s *t* test. †χ^2^test. ‡Fisher’s exact test. PG-SGA SF, Patient-Generated Subjective Global Assessment Short Form.

**Table 2:** Surgical details of patients with and without surgery-related muscle loss (SRML)

	Total (*n* = 173)	SRML (*n* = 68)	No-SRML (*n* = 105)	*P* value
**Type of tumour**				
ȃAppendix	2 (1.2)	0	2 (1.9)	
ȃBile ducts	31 (17.9)	12 (18)	19 (18.1)	
ȃColon	17 (9.8)	7 (10)	10 (9.5)	
ȃColorectal liver metastases	13 (7.5)	3 (4)	10 (9.5)	
ȃColorectal peritoneal metastases	6 (3.5)	2 (3)	4 (3.8)	
ȃLiver	10 (5.8)	4 (6)	6 (5.7)	
ȃPancreas	47 (27.2)	19 (28)	28 (26.7)	
ȃPseudomyxoma peritonei	4 (2.3)	1 (1)	3 (2.9)	
ȃRectum	24 (13.9)	15 (22)	9 (8.6)	
ȃSmall bowel	4 (2.3)	1 (1)	3 (2.9)	
ȃOther	15 (8.7)	4 (6)	11 (10.5)	
**Type of operation**				
ȃLiver segment resection	42 (24.3)	13 (19)	29 (27.6)	
ȃColorectal resection	36 (20.8)	21 (31)	15 (14.3)	
ȃPancreatic resection	64 (37.0)	26 (38)	38 (36.2)	
ȃCRS with HIPEC	22 (12.7)	7 (10)	15 (14.3)	
ȃNon-therapeutic laparotomy	7 (4.0)	2 (3)	5 (4.8)	
ȃOther	3 (1.7)	0	3 (2.9)	
ȃOperation time (min)	448 (170)	460 (172)	426 (169)	0.456*
ȃBlood loss (ml)	500 (277–1200)	525 (300–1375)	500 (225–1050)	0.205†
ȃCCI complications	20.9 (0–29.6)	12.25 (8.7–30.9)	12.24 (0–30.0)	0.260†
ȃSevere complications, Clavien-Dindo Grade ≥III ȃ(within 1 week postoperatively)	23 (13.3)	13 (19)	10 (9.5)	0.069‡
ȃSevere complications, Clavien-Dindo Grade ≥III ȃ(during full postoperative course)	35 (20.2)	16 (24)	19 (18.1)	0.385‡
ȃRelaparotomy	21 (12.1)	10 (15)	11 (10.5)	0.405‡
ȃIn-hospital mortality	2 (1.2)	2 (3)	0 (0)	0.153§
ȃDuration of ICU admission (days)	1 (0–1)	1 (0–1)	1 (0–1)	0.142†
ȃLength of hospital admission (days)	12 (9–19)	13 (10–20)	12 (9–19)	0.475†
ȃ30-day readmission	29 (16.8)	11 (16)	18 (17.1)	0.868‡

Data are presented as mean (s.d.), median (interquartile range), or *n* (%). *Student’s *t* test. †χ^2^ test. ‡Mann–Whitney *U* test. §Fisher’s exact test. CRS, cytoreductive surgery; HIPEC, hyperthermic intraperitoneal intraoperative chemotherapy; CCI, Comprehensive Complication Index.

Mean age was 64.3 (11.9) years and did not differ between patients with or without SRML. The SRML group consisted of a higher number of males than the non-SRML group (44 men (65 per cent) *versus* 51 men (49 per cent); *P* = 0.037). Mean preoperative BMI in the total cohort was 26.2 (4.8) kg/m^2^, and a total of 19 patients (28 per cent) with SRML suffered from 10 per cent weight loss or more in the 6 months prior to surgery *versus* 15 patients (14 per cent) without SRML (*P* = 0.087) (*Table 1*).

### Surgical details

Mean operating time was 448 (170) min and did not differ between the two groups. A trend towards higher severe complication rates within the first postoperative week in the SRML group was observed (13 (19 per cent) *versus* 10 (10 per cent); *P* = 0.069). From index surgery to discharge after unplanned readmissions, no differences in the median (i.q.r.) number of complications, according to CCI, were seen (12.3 (8.7–30.8) with SRML *versus* 12.2 (0–29.6) without SRML; *P* = 0.260). Median duration of hospital stay did not differ between the two groups (*Table 2*).

### Postoperative physical activity and nutritional intake

**Fig. 2:**
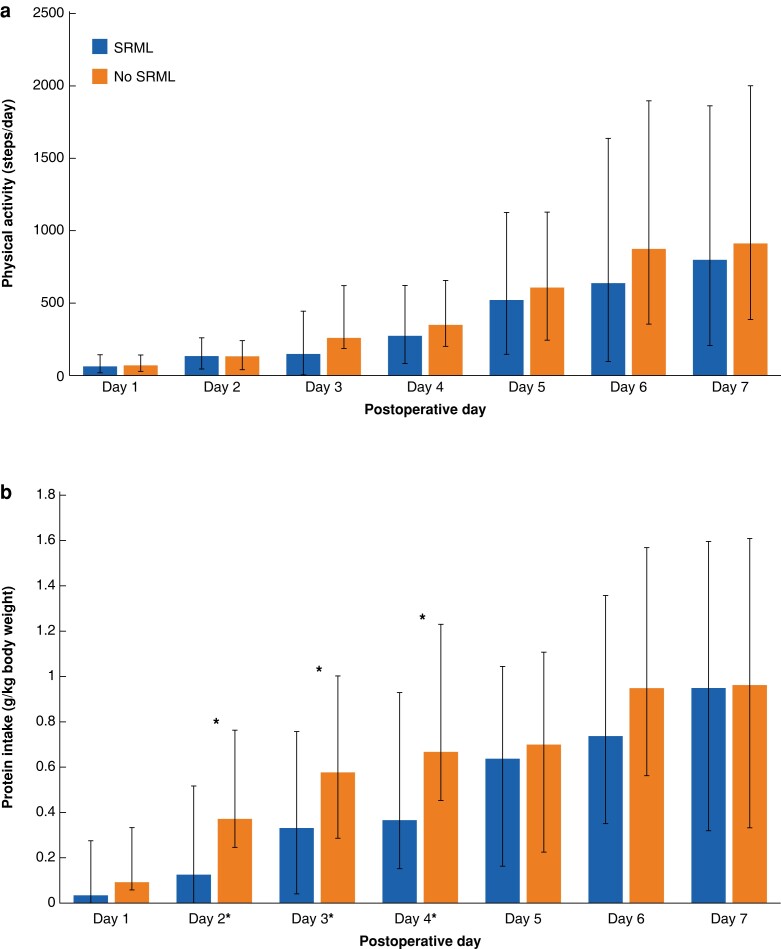
Physical activity and nutritional intake on each postoperative day after major abdominal surgery **a** Physical activity in median (interquartile range (i.q.r.)) steps taken per day postoperatively, compared between patients with and without surgery-related muscle loss (SRML). **b** Nutritional intake in median (i.q.r.) grams of protein per kilogram of body weight per day postoperatively, compared between patients with and without SRML. *P <0.05 in intake, compared between patients with and without SRML (Mann–Whitney *U* test).

Daily increases in physical activity and protein intake were observed in both groups (*Fig. 2*). No differences were seen in steps taken daily. The median total number of steps taken over 5 days did not differ between patients with or without SRML (1327 (532–2368) steps *versus* 1603 (703–2657) steps, respectively; *P* = 0.592). For the patients with available data on physical activity, insufficient physical activity was observed in 21 out of 52 patients (40 per cent) with SRML and 26 out of 80 patients (33 per cent) without SRML (*P* = 0.355).

Regarding nutritional intake, statistically significant differences were observed between the SRML and non-SRML group at postoperative days 2, 3, and 4 (*P <* 0.050) (*Fig. 2b*). After five postoperative days, the cumulative median protein intake of patients with SRML was significantly lower compared with patients without SRML (1.35 (0.058–3.33) g/kg *versus* 2.95 (0.99–4.83) g/kg; *P* = 0.009). Nutritional intake in 64 of 152 patients (42 per cent) was insufficient (30 out of 60 for SRML (50 per cent) *versus* 34 out of 91 for non-SRML (37 per cent); *P* = 0.111).

When assessing the combination of insufficient physical activity and insufficient nutritional intake, a significant difference was seen between patients with sufficient physical activity and sufficient nutritional intake (13 out of 46 with SRML (38 per cent) *versus* 34 out of 77 without SRML (44 per cent)), patients with only one sufficient (23 out of 46 with SRML (50 per cent) *versus* 38 out of 77 without SRML (49 per cent)) and patients with both insufficient physical activity and insufficient nutritional intake (10 out of 46 with SRML (22 per cent) *versus* 5 out of 77 without SRML (6 per cent)) (*P* = 0.025).

### Risk factors for surgery-related muscle loss

A univariate and multivariable logistic regression model with factors associated with clinically relevant SRML is presented in *Table 3*. Multivariable logistic regression analysis showed a significantly increased risk of developing SRML in patients with 10 per cent or more weight loss within 6 months preoperatively (odds ratio (OR) 3.89, 95 per cent c.i. 1.35 to 11.06; *P* = 0.012), and in patients who had both insufficient postoperative physical activity and insufficient nutritional intake (OR 4.00, 95 per cent c.i. 1.03 to 15.47; *P* = 0.045) (*Table 3*). An increased intake of proteins (g/kg body weight) was associated with a lower risk of developing SRML (OR 0.78, 95 per cent c.i. 0.65 to 0.93; *P* = 0.007). A trend towards an increased risk of patients aged 65 years or older of developing SRML was seen (OR 2.39, 95 per cent c.i. 1 to 5.70; *P* = 0.050).

**Table 3:** Univariate and multivariable logistic regression analyses for factors associated with clinically relevant surgery-related muscle loss after major abdominal surgery

		Univariate analysis			Multivariable analysis		
Factor	*n*	OR	95% c.i.	*P* value	aOR*	95% c.i.	*P* value
**Age**							
ȃ<65	72	1			1		
ȃ≥65	101	1.39	0.75–2.61	0.298	2.39	1–5.70	**0** .**050**
**Preoperative weight loss**							
ȃ<5%	111	1			1		0.038
ȃ5–10%	28	1.03	0.43–2.44	0.954	1.06	0.36–3.13	0.913
ȃ>10%	34	2.34	1.07–5.11	**0** .**033**	3.87	1.35–11.06	**0** .**012**
**Diabetes mellitus**							
ȃNo	152	1			1		
ȃYes	21	0.58	0.21–1.58	0.286	0.60	0.18–2.02	0.604
**Severe complications, Clavien–Dindo grade ≥III within 1 week postoperatively**							
ȃNo	150	1			1		
ȃYes	23	2.25	0.92–5.46	0.074	1.01	0.30–3.39	0.985
					aOR*		
**Cumulative physical activity in 5 days (no. of steps)**	132	1	1–1	0.699	1	1–1	0.919
ȃMissing	41						
**Cumulative intake in 5 days (g protein/kg)**	152	0.82	0.70–0.95	**0** .**009**	0.78	0.65–0.93	**0** .**007**
ȃMissing	21						
					**aOR** †		
**Insufficient physical activity and nutritional intake for ≥3 days in the first week‡**							
ȃBoth sufficient	47	1			1		0.129
ȃOne sufficient	61	1.49	0.65–3.41	0.345	1.30	0.54–3.07	0.566
ȃBoth insufficient	15	4.92	1.41–17.22	**0** .**013**	4.00	1.03–15.47	**0** .**045**
ȃMissing	50						

Adjusted odds ratio (aOR): logistic regression model adjusted for age 65 years or older, preoperative weight loss, (preoperative) diabetes mellitus, severe complications within 1 week postoperatively, cumulative intake in 5 days, cumulative physical activity in 5 days. †Adjusted odds ratio (aOR): logistic regression model adjusted for age 65 years or older, preoperative weight loss, (preoperative) diabetes mellitus, severe complications within 1 week postoperatively, and insufficient nutritional intake and physical activity. ‡Insufficient physical activity and nutritional intake was defined as a physical activity (steps taken daily) or nutritional intake (g/kg body weight) below the lowest tertile for at least 3 days during the first postoperative week.

### Postoperative fatigue

No significant differences between fatigue in patients with or without SRML were observed at baseline, 3 months postoperatively, and 6 months postoperatively (*Table 4*).

**Table 4:** Multidimensional Fatigue Inventory score at baseline, 3 months postoperatively, and 6 months postoperatively in patients with and without surgery-related muscle loss (SRML)

	Baseline			3 months postoperatively			6 months postoperatively		
	SRML (*n* = 68)	No SRML (*n* = 105)	*P* value	SRML (*n* = 68)	No SRML (*n* = 105)	*P* value	SRML (*n* = 68)	No SRML (*n* = 105)	*P* value
Total score	20.5 (7.25–31)	21 (5.25–31.75)	0.757*	24 (15–41.75)	32 (20–42)	0.167*	18 (7–33)	25 (11–37)	0.069*
Physical subscale	10 (3–15)	9.5 (2.25–16)	0.984*	13 (9–20)	16 (10–22)	0.235*	9 (3–17.5)	13 (7–20)	0.082*
Cognitive subscale	8.5 (1–12.25)	7.5 (1.25–14.75)	0.812*	8.5 (2–17)	12 (5–17)	0.205*	6 (0.25–11.75)	9 (2–16)	0.093*
Psychosocial subscale	2 (0–4)	2 (0–4)	0.784*	3 (2–5)	3.5 (2–5)	0.635*	2 (0.25–3)	3 (1–4)	0.108*
Missing (%)	4 (6)	1 (1.0)		20 (29)	22 (21.0)		28 (41)	26 (24.8)	

Values are median (interquartile range) unless otherwise indicated. A lower score defines a more favourable health state. *Mann–Whitney *U* test.

### One-year survival

**Fig. 3:**
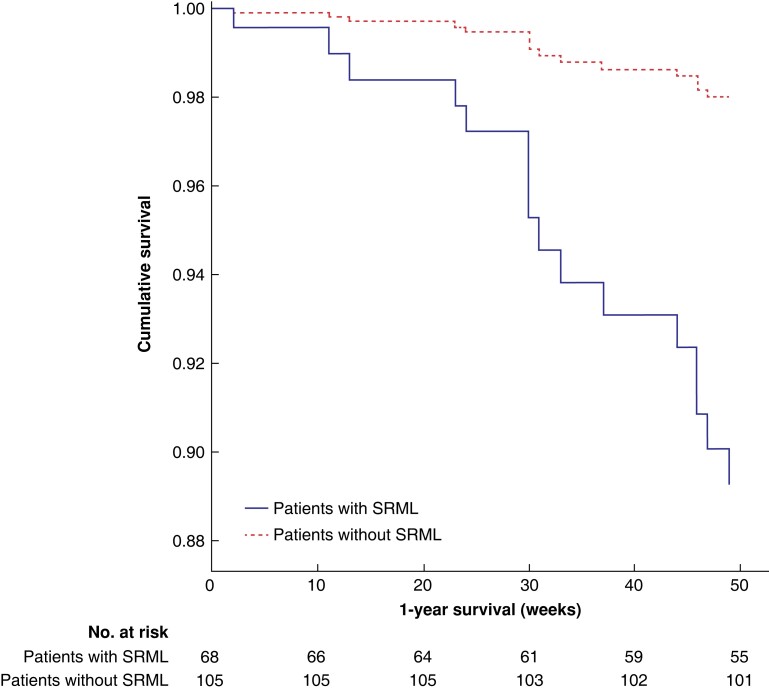
Multivariable Cox proportional hazards regression analyses of 1-year survival after major abdominal surgery compared between patients with and without surgery-related muscle loss Multivariable Cox proportional hazards regression analyses for factors associated with 1-year survival after major abdominal surgery grouped by the presence of surgery-related muscle loss (SRML) within 1 week postoperatively, as shown in *Table 4*. The multivariable Cox proportional hazard regression analysis was adjusted for age 65 years or older, type of surgery, surgical R status, and postoperative complications according to the Comprehensive Complication Index.

Univariate and multivariable Cox proportional hazards regression analyses on 1-year survival after major abdominal cancer surgery are presented in *Table 5*. Factors influencing 1-year survival in the univariate analysis were R2 resection status (hazard ratio (HR) 5.21, 95 per cent c.i. 1.14 to 23.81; *P* = 0.033), postoperative complications in CCI (HR 1.03, 95 per cent c.i. 1.01 to 1.06; *P* = 0.002), and the presence of SRML (HR 5.43, 95 per cent c.i. 1.77to 16.65; *P* = 0.003). Multivariable analysis, adjusted for age, type of surgery, resection status, and postoperative complications, identified clinically relevant SRML as a factor independently associated with decreased 1-year survival (HR 5.11, 95 per cent c.i. 1.61 to 16.21; *P* = 0.006) (*Table 5*, *Fig. 3*).

**Table 5:** Univariate and multivariable Cox proportional hazards regression analyses for factors associated 1-year survival after major abdominal surgery

Factor	Univariate analysis			Multivariable analysis		
	HR	95% c.i.	*P* value	HR	95% c.i.	*P* value
Age ≥65 years	3.45	0.99–12.00	0.052	3.81	0.83–17.51	0.085
**Type of surgery (ITT)**						
ȃLiver	1		0.326	1		0.122
ȃPancreatic surgery	0.611	0.21–1.74	0.356	0.36	0.11–1.17	0.090
ȃColorectal surgery	0.154	0.019–1.249	0.080	0.14	0.02–1.18	0.070
ȃCRS + HIPEC	0.530	0.100–2.551	0.428	0.23	0.03–1.92	0.174
R-status *						
ȃR0 resection	1		0.065	1		**0**.**014**
ȃR1 resection	1.99	0.625–6.35	0.244	3.08	0.87–10.94	0.082
ȃR2 resection	5.213	1.14–23.81	**0** .**033**	11.32	2.01–63.87	**0** .**006**
ȃCCI complications	1.033	1.012–1.055	**0** .**002**	1.03	1.01–1.05	**0** .**011**
ȃSRML present	5.429	1.77–16.654	**0** .**003**	5.11	1.61–16.21	**0** .**006**

R-status indicates the surgical resection status: R0 indicates a microscopically margin-negative resection; R1 indicates the removal of all macroscopic disease but with microscopic-positive tumour margins; R2 indicates a macroscopic residual tumour that was not resected. HR, hazard ratio; ITT, intention to treat; CCI, Comprehensive Complication Index; SRML, surgery-related muscle loss of at least 10% in one arm and one leg muscle within 1 week postoperatively.

## Discussion

The results of this study demonstrated that 39 per cent of patients suffered from clinically relevant SRML within 1 week of major abdominal cancer surgery. It also revealed that a combination of insufficient postoperative physical activity and nutritional intake formed an independent risk factor for developing SRML. Additionally, clinically relevant SRML was independently associated with decreased 1-year survival after major abdominal cancer surgery.

The association between preoperative sarcopenia with worse postoperative outcomes has been studied extensively^18–22^. Researchers hypothesize that skeletal muscles are essential for systemic metabolism and are, aside from their function in locomotion, fundamental to systemic metabolism and energy homeostasis^23,24^. Factors with an influence on preoperative sarcopenia are ageing, malnutrition, physical inactivity, chronic disease, and cancer cachexia^21^. However, for the postoperative patient, surgical trauma seems to play a key role in the catabolic response, resulting in accelerated postoperative muscle loss^25,26^. Therefore, in this study we focused on this specific phase of acute muscle loss measured by bedside POCUS in the surgical patient. POCUS is a reliable and validated imaging technique for measuring skeletal muscle, so we were able to measure at multiple time points within the acute postoperative phase without exposing patients to additional ionizing radiation^27–33^.

Few studies have focused on acute surgical muscle loss and its risk factors in this early postoperative phase^5,9,11^. Van Wijk *et al*. demonstrated that SRML, measured with

CT, occurred in 67 of 128 patients (52.3 per cent) within 1 week of liver resection^5^, and Huang *et al*. identified SRML in 35 of 110 patients (31.8 per cent) within 1 week of gastrectomy^11^. Similarly to our results, van Wijk *et al*. and Huang *et al*. both observed an association between being aged 65 years or older and clinically relevant SRML^5,11^. This suggests that the muscles of elderly people are more prone to muscle protein breakdown or have slower muscle protein synthesis than younger people, or that they are affected by a combination of these two factors^34^. Other factors that these studies found to be associated with SRML were the presence of pulmonary comorbidities and diabetes mellitus; however, these were not of influence in the current study. However, unlike previous studies, the present study included postoperative physical activity and nutritional intake measurements in the assessment of SRML, creating a more complete model of modifiable factors influencing muscle loss. Regarding muscle loss and postoperative complications, similar to previous studies, a trend towards more severe complication rates in the first week in patients with SRML was observed^5,9,11^. However, it is difficult to establish a causal relationship between muscle loss, complications, and lack of appetite. However, when assessing possible preventable risk factors for SRML, the strong association between protein intake and muscle loss (adjusted for complications) could be an important lead in the prevention of skeletal muscle loss.

This study emphasizes, in particular, the relevance of the prevention of muscle loss when the impact of SRML on survival is considered. Previous studies have also reported associations between muscle loss and long-term survival^6,10,35,36^. For example, the study conducted by Argillander *et al*. (with 233 patients) demonstrated that patients with muscle loss 1 year postoperatively had a 2.7-fold higher mortality risk than those without^35^. Furthermore, other studies—where muscle loss was measured several months postoperatively—found that muscle loss was associated with poorer long-term survival^6,10,36^. However, this is the first study to show that muscle loss occurring within 1 week of surgery is an independent risk factor for decreased survival. Since this impact of muscle loss on survival has also been seen in non-surgical patients admitted to the ICU^37,38^, or in patients undergoing chemotherapy^39^, the loss of muscle mass is an important issue applicable to all patients. An additional consequence of muscle loss found by Huang *et al*. (110 patients) is prolonged fatigue months after surgery^11^. However, unlike this previous study, we did not find this association. A possible explanation for these contradictory results could have been the result of bias in our study due to patients who did not respond to our follow-up questionnaires after 3 and 6 months (24 per cent and 31 per cent, respectively); patients with higher levels of fatigue may have been less likely to respond to the questionnaires than those with lower fatigue.

By identifying risk factors such as preoperative weight loss, nutritional intake, and physical activity, this study has established some leads for the prevention of SRML in clinical practice. For example, patients with severe weight loss in the preoperative phase could be included in prehabilitation programmes providing support such as physiotherapy and dietician consultations prior to surgery^40^. Furthermore, education on the importance of artificial nutrition in the preoperative phase could lead to an increased drive for patients to improve both pre- and postoperative intake.

Notably, previous studies have illustrated that providing sufficient nutritional support for surgical patients in the early postoperative phase is difficult^41–43^. Despite existing guidelines from the European Society of Parenteral and Enteral Nutrition stating that the minimal postoperative protein and energy requirements for maintaining muscle mass are 1.5 g of protein and 25–50 kcal per kg of body weight per day, respectively, postoperative discomfort and healthcare-related factors—such as missed meals and a lack of focus on nutrition—are still barriers to the adequate provision of nutrition^26,44,45^. Therefore, since this study demonstrated that nutritional deficiency in the days immediately following surgery is highly associated with SRML, it is our opinion that direct intraoperative placement of nasojejunal feeding tubes could be the solution during the first postoperative days in patients undergoing major abdominal surgery. However, this goes against the current guidelines of enhanced recovery after surgery (ERAS) programmes, which focus on early reduction of drains and tubes (for example, epidural pain management and nasogastric tubes) to enhance early mobilization and oral intake^46–49^. In our opinion, the magnitude of these specific major abdominal surgeries is possibly too large for patients to properly implement some aspects of ERAS programmes for this patient group. Therefore, we recommend adjusted ERAS protocols with an increased focus on nutritional support by means of nasojejunal feeding tubes and protein-enriched products from the first postoperative day onward.

To our knowledge, this is the first study to investigate surgery-related changes in muscle thickness and their association with physical activity and nutritional intake; however, this study has certain limitations. The first was due to the gathering of peripheral oedema between the subcutis and muscle fibres of the upper legs pelvis and in the first postoperative days as a result of the fluid shifting induced by the surgical stress response^1,2,50^. Because of this, increased measured muscle thickness could possibly have resulted in the underestimation of the group of patients with SRML. Furthermore, because patients who underwent surgery for various oncological indications were included, it is difficult to provide disease-specific analyses of risk factors and the impact of SRML. Nonetheless, this prospectively conducted study included data on muscle loss, surgical details, postoperative physical activity, and nutritional intake of 173 patients who underwent major abdominal cancer surgery; it, therefore, had enough power to detect potential leads for further disease-specific research in the prevention of postoperative muscle loss.

In conclusion, we found that clinically relevant SRML occurred in 39 per cent of patients after major abdominal cancer surgery and was significantly associated with decreased 1-year survival. Risk factors found to be associated with SRML were preoperative weight loss and a combination of insufficient physical activity and insufficient nutritional intake within the first week postoperatively. Therefore, to reduce muscle loss, pre- and postoperative programmes focused on adequate protein intake and early mobilization should play a key role in surgical oncological treatment.

## Data Availability

The data sets generated or analysed in the current study are not publicly available as the data are linked to a vulnerable patient population, but they are available from the corresponding author upon reasonable request.
